# Microglial inhibition of neuroprotection by antagonists of the EP1 prostaglandin E2 receptor

**DOI:** 10.1186/1742-2094-6-5

**Published:** 2009-02-17

**Authors:** Noel G Carlson, Monica A Rojas, John-David Black, Jonathan W Redd, John Hille, Kenneth E Hill, John W Rose

**Affiliations:** 1 Geriatric Research Education and Clinical Center (GRECC), VASLCHCS, Salt Lake City, UT 84148, USA; 2 Neurovirology Research Laboratory, VASLCHCS, Salt Lake City, UT 84148, USA; 3 Department of Neurobiology and Anatomy, University of Utah, Salt Lake City, UT 84112, USA; 4 Department of Neurology, University of Utah, Salt Lake City, UT 84112, USA; 5 Brain Institute, University of Utah, Salt Lake City, UT 84112, USA; 6 Center on Aging, University of Utah, Salt Lake City, UT 84112, USA

## Abstract

**Background:**

The EP1 receptor for the prostanoid PGE2 is a G-protein coupled receptor that has been shown to contribute to excitotoxic neuronal death. In this study we examined the influence of non-neuronal cells on neuroprotective properties of EP1 receptor antagonists (Ono 8711 and SC 51089).

**Methods:**

Primary neuronal cultures systems with or without non-neuronal cells were used to examine how the neuroprotective properties of EP1 antagonists were influenced by non-neuronal cells. The influence of astrocytes or microglia were individually tested in excitotoxicity assays using a co-culture system with these cells grown on permeable transwell inserts above the neuronal-enriched cultures. The influence of microglia on PGE2 synthesis and EP1 receptor expression was examined.

**Results:**

EP1 antagonists were neuroprotective in neuronal-enriched cultures (> 90% neurons) but not in mixed cultures (30% neurons plus other non-neuronal cells). Co-cultures of microglia on permeable transwell inserts above neuronal-enriched cultures blocked neuroprotection by EP1 antagonists. Incubation of microglia with neuronal-enriched cultures for 48 hours prior to NMDA challenge was sufficient to block neuroprotection by EP1 antagonists. The loss of neuroprotection by EP1 antagonists was accompanied by a decrease of neuronal EP1 expression in the nucleus in cultures with microglia present.

**Conclusion:**

These findings demonstrate microglial modulation of neuronal excitotoxicity through interaction with the EP1 receptor and may have important implications in vivo where microglia are associated with neuronal injury.

## Background

Cyclooxygenase-2 (COX-2), the enzyme that catalyzes the rate limiting step in the synthesis of prostanoids, contributes to neuronal death. Inhibitors of COX, termed non-steroidal anti-inflammatory drugs (NSAIDs) [1], can protect neurons following an assault with toxic stimuli that promote excitotoxic death; both in vitro [2,3] and in vivo [4-7]. COX-2 knockout mice are also less susceptible to excitotoxicity following exposure to the glutamate receptor agonist N-methyl D-aspartate (NMDA) [8]. Therefore, a loss of COX-2 activity either by inhibition of the enzyme or loss of expression is associated with increased neuronal viability. Conversely, increased COX-2 activity appears to augment neuronal death. The increased COX-2 expression in neurons observed in vivo in animal models of stroke [4], following stimulation with the glutamate receptor agonist kainic acid [6], and in vitro following NMDA stimulation [2,3] is coincident with loss of neurons. Constitutive expression of COX-2 in neurons at high amounts in transgenic mice results in a greater loss of neurons in stroke models [9] and age-associated loss of neurons [10]. In addition, constitutive COX-2 expression renders neurons more susceptible to NMDA-stimulated death [11].

There are two COX genes, COX-1 and COX-2 [1]. COX catalyzes the initial steps in the conversion of arachidonic acid (AA) to one of the five prostanoids, prostacyclin (PGI2), thromboxane (TxA2), prostaglandin D2 (PGD2), prostaglandin F2α (PGF2α) and prostaglandin E2 (PGE2) [1,12]. In addition to the generation of prostanoids, reactive oxygen species (ROS) are also generated by COX-2 in the reaction of prostanoids [1]. It was demonstrated that the COX-2-generated prostanoids (and not ROS), are the major contributors by COX-2 towards excitotoxicity following administration of NMDA to animals [13].

Each of the prostanoids synthesized by COX activates at least one specific prostanoid receptor. These receptors are coupled to G-proteins and are designated IP (for PGI2), TP (for TXA2), DP1 or DP2 (for PGD2), FP (for PGF2α) and EP1-4 (for PGE2) [12]. Recent investigations have focused on understanding how activation of specific prostanoids affects neuronal viability. In our earlier studies we identified that PGF2α and PGE2 were made in primary neuronal cortical cultures in response to stimulation with NMDA [3,14]. An analog of PGE2, 17-phenol trinor PGE2 (17-pt-PGE2), but not PGF2α, could reverse the neuroprotective effect of a COX-2-specific inhibitor in vitro [3] and in vivo [13] following NMDA administration. These studies indicate that PGE2 production by COX-2 can contribute to the deleterious actions of COX-2 in NMDA-mediated excitotoxicity of neurons. However, in vitro studies investigating the role of PGE2 and its analogs have yielded contradictory results. PGE2 or its analogs have been reported to both increase neuronal survival following NMDA stimulation [15-19] and in some cases be neurotoxic [20,21]. These opposing effects or PGE2 on neuronal viability are due to activation of specific EP receptors that exert either pro survival or pro death effects. In general, activation of EP1 contributes to neuronal death [21-24], while activation of EP2 [17-19] and EP4 [24] promote neuroprotection. EP1 has been shown to contribute to NMDA-mediated neuronal death in vivo [24]. Decreased EP1 activation by a pharmacologic antagonist or genetic knockout of the EP1 receptor decreased NMDA-stimulated neuronal death, whereas a specific EP1 receptor agonist augmented death [22-24].

Significant progress has been made in understanding how prostanoids contribute to neuronal death [25]. EP1 receptor activation in neurons has been linked to two different intracellular mechanisms tied to excitotoxic cell death. EP1 receptor activation was initially shown to impair the Na^+^-Ca^2+ ^exchanger (NCX) which subsequently causes greater increases in intracellular calcium leading to neuronal death [23]. More recently, EP1 receptor activation has been linked to the AKT signaling pathway that affects neuronal viability [26].

However, the interaction of EP1 with other cell types in the central nervous system (CNS) is not well understood. In this study, we examined whether inhibition of neuronal EP1 contributes to neuronal viability in primary cultures with differing compositions of non neuronal CNS cells. We investigated the neuroprotective properties of two specific EP1 receptor antagonists, Ono 8711 [27] and SC51089 [28] along with a COX-2 inhibitor NS398. This study demonstrates that the presence of microglia in culture can offset the neuroprotective effects of EP1 antagonists. Our findings have important implications to conditions where microglia may accumulate following neuronal injury and potentially limit neuroprotection conferred by EP1 antagonists.

## Methods

### Reagents and antibodies

Ono 8711 was generously provided by Takayuki Maruyama Ph.D., Ono Pharmaceutical Company, (Osaka, Japan). N-[2-(cyclohexyloxy)-4-nitrophenyl]-methanesulfonamide (NS398) was obtained from Cayman Chemical Company, (Ann Arbor MI). SC51089 was obtained from Biomol, (Plymouth Meeting, PA). NMDA, MK801 and Arabinose cytosine (AraC) were obtained from Sigma Chemical Company (St. Louis, MO). Tissue culture media minimum essential media (MEM) was obtained from Sigma Chemical Company (St. Louis, MO), glutamine and trypsin were from Gibco Life Sciences, Invitrogen (Carlsbad, CA). Serum (defined fetal bovine serum and defined horse serum) was purchased from Hyclone (Logan, UT). Primary antibodies used in this study were, mouse monoclonal anti microtubule associated protein (MAP)-2 (BD Pharmingen, San Diego, CA), rabbit polyclonal EP1 (Cayman Chemical Company), rat monoclonal CD11b (Abcam, Cambridge, MA), rat monoclonal anti glial fibrillary acidic protein (GFAP)(Calbiochem, San Diego, CA) and rat monoclonal anti mouse F4/80 was produced by our laboratory. Secondary antibodies used included FITC-coupled goat anti-mouse, Cy5-coupled anti-rat and Cy5-coupled goat anti-rabbit. Secondary antibodies were obtained from Jackson Immuno-Research Laboratories Inc. (West Grove, PA). Propidium iodide (PI) was obtained from Molecular Probes (Eugene, OR).

### Primary cortical cultures

Cortical cultures were prepared essentially as described previously [3,29-31]. Cortices from E15 embryos (mouse strain CD1, from Charles River Laboratories, Wilmington MA) were removed and the cells were dissociated by digestion with trypsin (0.025%) for 30 min at 37°C, followed by physical dissociation resulting from pipeting the cells (15–20 times) through a 5-ml pipette placed in a 15-ml conical tube with a loose seal between the bottom of the tube and the pipette. The cells were then plated at a density of 1.1 × 10^6 ^trypan blue-excluding cells per well in a 12-well plate (Corning dishes pre coated with poly-D-lysine). Cells were initially plated in 1-ml of MEM with Earle's salts supplemented with 5% horse serum (heat inactivated), 5% fetal bovine serum (heat inactivated), 30 mM glucose, 2 mM glutamine and grown at 37°C in humidified chambers with 5% CO_2_. The cultures were fed the next day by replenishing about half the volume with fresh growth media (MEM with Earle's salts supplemented with 10% horse serum, 30 mM glucose, 2 mM glutamine) and fed every 2–3 days after that to generate mixed cortical cultures. After nine days in culture, these mixed cultures were exposed to AraC (4–8 μM) for a 24 hour period to limit the growth of mitotic cells. Each preparation of AraC differed with respect to its anti-mitotic efficacy and was tested for optimal inhibition of cell growth with minimal toxicity. These mixed culture preparations contained approximately 30% of the cells as neurons [3]. All excitotoxicity experiments with mixed cultures were started 2 days after the last feeding and were used 15 days after plating. At this stage, the neurons exhibited the most consistent response to NMDA while still having low background death. Neuronal-enriched cultures were prepared as above except that, on the second day after plating, the cells were treated with 2–4 μM AraC for a 24-hour period and fed only once more with feeding media (MEM with Earle's salts supplemented with 10% horse serum, 30 mM glucose, 2 mM glutamine). Neuronal-enriched cultures which were incubated for 8 days in vitro (DIV), contained between 90–95% neurons as determined by immunofluorescent microscopy as the number of PI stained nuclei that were associated with cells labeled with the neuronal-specific marker MAP-2 (data not shown). Excitotoxicity experiments with these cultures were started after the cultures were maintained for 8 DIV, a time at which there is low background neuronal death and the neurons exhibit a consistent response to NMDA stimulated excitotoxicity. Excitotoxicity experiments were performed in growth media that contained 10% horse serum unless stated otherwise.

Cortical cultures were also examined in serum-free media. Cortical cultures were prepared as described and allowed to incubate in plating media (MEM with Earle's salts supplemented with 5% horse serum, 5% fetal bovine serum, 30 mM glucose, 2 mM glutamine) for 24 hours. The media was then replaced with neurobasal media containing 2% B27 supplement (Invitrogen) and, 24 hours later, the cultures were then exposed to AraC (4–8 μM). The cultures were fed 24 hours after this by replacing one half of the media with fresh Neurobasal media with 2% B27 supplement. These cultures were used in excitotoxicity experiments 8 days after the initial plating.

### Excitotoxicity assay

**Figure 1:**
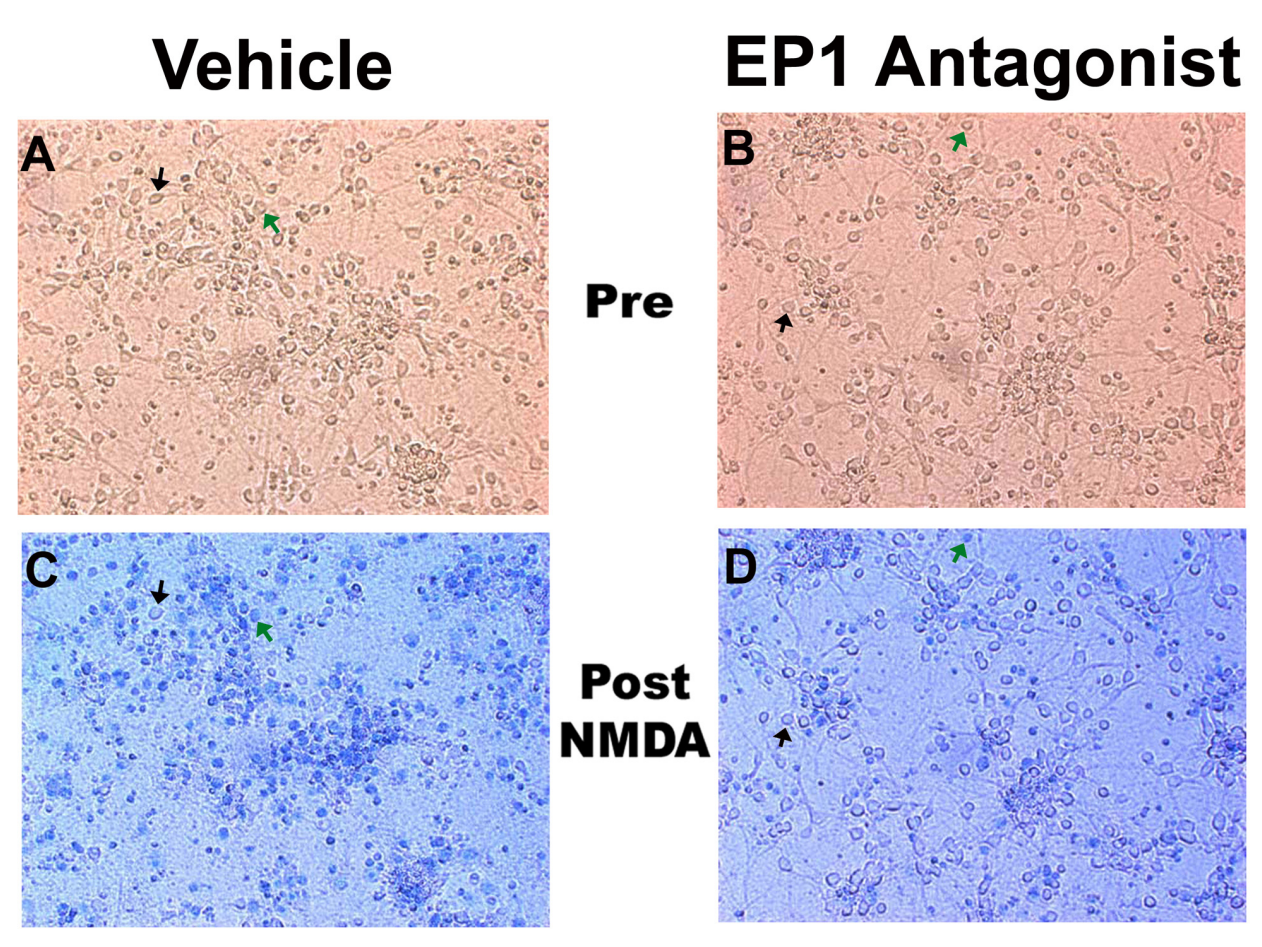
**Excitotoxicity assay and neuroprotection by Ono 8711**. Fields of neuronal-enriched cultures were photographed before treatment with vehicle (A) or the EP1 receptor antagonist Ono 8711 (B). These cultures were then treated with NMDA (15 μM) and photographed twenty hours later after staining with trypan blue dead cell stain for vehicle (C) and Ono 8711 (D). Examples of cells that survived NMDA treatment and excluded trypan blue are indicated by a black arrow in each panel. An example of a cell in each field that was killed by NMDA (as indicated by staining with trypan blue) is indicated by a green arrow. The numbers of neurons were counted for each of the cultures before and after NMDA (as non Trypan-stained cells). In this example, the addition of 30 nM Ono 8711 increased the percent of surviving neurons from 20% to 50%. Images were captured at 20 × magnification.

Excitotoxicity was measured using a modified protocol of established methods as described in detail elsewhere [3,29-31]. Neuronal cell death was assessed by photographing the same four fields in each well, before and after addition of NMDA. Each field contained 150 to 300 neurons prior to treatment with NMDA. The numbers of intact neurons were counted before NMDA treatment as the phase bright cells (see Figure 1). Twenty hours after treatment with NMDA (in the presence or absence of neuroprotective agents) one ml of trypan blue (0.4%) was added to the media (final concentration of 0.2%) and the volume of this mixture was adjusted to leave minimal solution to cover the cells. The cells were incubated with the trypan solution for 20 minutes. Plates were marked with reference so that the same fields could be photographed as shown in Figure 1. Cells that did not stain with trypan blue were counted as live cells. These trypan blue-excluding cells also did not stain by the necrotic dead cell stain ethidium homodimer (Molecular Probes), but did stain with the live cell stain Calcein AM green (Molecular Probes) (data not shown). All excitotoxicity experiments were repeated at least three times. The percentages of surviving neurons were determined in each field by dividing the total of trypan-excluding cells after NMDA treatment by the total number of cells in that field counted prior to NMDA. A total of three fields were examined in each treatment per plate. The average percent neuronal survival was assessed for each treatment from at least three independent experiments and included the average from a minimum of nine fields for each condition reported in each figure. In mixed cultures, neurons were identifiable as phase-bright cells that were observed in a focal plane above the glial monolayer. This was confirmed in sister cultures by immunofluorescence showing that these cells were stained with the neuronal marker MAP-2 [3,29-31].

The cell counting method for determining viable cells can be used when cultures are grown in the presence of serum (as in most of our current experiments) or when testing agents that may have antioxidant properties which interfere with assays that measure release of the enzyme lactate dehydrogenase (LDH) into the media from dead cells. For each experiment, the concentration of NMDA required to give about 80% neuronal death was pre-determined and varied between different lots of NMDA and cell preparations. Typical concentrations of NMDA required for 80% neuronal death ranged between 10–40 μM for neuronal-enriched cultures and 15–80 μM for mixed cultures. All experiments were conducted using this high amount of NMDA-induced neuronal death for convenience and reliability in scoring the survival after NMDA treatment. For neuroprotective experiments, the neuroprotective agent (NS398, Ono 8711 or SC51089) was added directly to the growth media coincident with the addition of NMDA. In all cases, the effect of each neuroprotectant treatment (or combination of treatments) on neuronal viability in the absence of NMDA was also determined by photographing the cells before and 20 hours after treatment with the agent indicated. In each experiment, 500 to 2000 neurons were counted for each treatment.

### Microglial and astrocyte cultures

Microglial cultures were prepared according to the methods described [32]. Brains were removed from 1 day old CD1 mice and the cerebral cortices were obtained. The meninges were removed and the tissue was minced and digested with trypsin (0.025%) for 30 min at 37°C, followed by physical dissociation as described earlier for neuronal cortical cultures. The cells were plated in MEM containing 10% fetal bovine serum into 25 cm^2 ^tissue culture flasks (1 brain per flask) that were pretreated with poly-d-lysine. Cultures were grown for 7–9 days at 37°C and the microglial cells were removed by shaking on an orbital shaker at 250 rpm for 30 min. Microglial cells were then centrifuged for 5 min at 1500 g and resuspended in neurobasal medium with 2% B27 supplement and plated onto either poly-D-lysine coated12-well plates or transwells (12 mm diameter) at a density ranging from 1.5 × 10^5 ^to 3 × 10^5 ^cells/mm^2^. Lower cell density microglial cultures were more difficult to grow. After 8 days in culture, microglial transwells were transferred onto 5 day old neuronal-enriched cultures (in 12 well plates) and co-cultured another 48 hours prior to the start of excitotoxicity experiments. Microglial cultures contained greater than 95% microglial cells as determined by staining with microglial markers, F4/80 or CD11b.

Astrocyte cultures were prepared as described [32]. Cells were isolated from cerebral cortices from 1 day old mice as described above and the cells were suspended in MEM containing 10% fetal bovine serum and plated onto either transwells or tissue cultures plates (1 × 10^5 ^cells/mm). These cultures were grown at 37°C for about 1–2 weeks until the cells reached confluency. Transwell astrocyte cultures were transferred to neuronal-enriched cultures (5 day old cultures), 48 hours before the start of excitotoxicity experiments.

### Conditioned media experiments

Media was harvested from mixed cultures (15 DIV) and neuronal-enriched cultures (8 DIV) and centrifuged for 5 minutes (1000 × g) to eliminate any residual cells. Freshly harvested media was subsequently exchanged between the mixed and neuronal-enriched culture preparations. Since the neuronal-enriched cultures (8 DIV) did not survive a direct media exchange, the mixed conditioned media was exchanged in the 12-well plates by seven two-fold dilutions. Neuronal-enriched media was directly exchanged on the mixed cultures (15 DIV) in one exchange. Excitotoxicity experiments were performed as described above.

### Transwell experiments

Mixed cortical cultures, microglia or astrocytes were plated onto the top side of the transwell inserts (0.4 μm pore size polycarbonate membrane coated with poly-D-lysine, Corning catalog number 3401, Corning, NY) at the cell density as described above. The mixed cultures on transwells were treated with AraC (on day 9) and fed as described for mixed cultures. These mixed culture transwells (15 DIV) were transferred to neuronal-enriched cultures 48 hours prior to treatment with NMDA. Neuronal-depleted mixed cultures were obtained by exposure of the mixed cultures (11 DIV) to 25 μM NMDA for 48 hours prior to transfer of the transwells to plates containing fresh media and three exchanges of media to remove NMDA. The transwell cultures were positioned approximately 2 mm above the neuronal-enriched cultures and the cells grown on the transwells were separated from the neuronal-enriched cultures by the permeable transwell membrane. The neuronal-enriched cultures were photographed prior to addition of the transwells and treatment with NMDA. The mixed culture transwells were then returned to the same wells of the neuronal-enriched cultures and subsequently treated with vehicle, SC51089, and then NMDA at a concentration that yielded approximately 20% neuronal survival. All drugs were added to the media below the transwells. After 24 hours, the mixed culture transwells were removed and the neuronal-enriched cultures were stained with trypan blue, photographed and scored for neuronal survival as described above. Viability of the mixed cultures, microglia cultures and astrocyte coultures on the transwells prior to NMDA treatment was verified using dead cell stain ethidium homodimer and the live cell stain Calcein AM green.

### Statistical analyses

The data from the NMDA excitotoxicity experiments were analyzed using InStat software (GraphPad software, San Diego, CA). Data from each treatment group passed the method of Kolmogorov and Smirnov for normal distributions making it appropriate for parametric tests to assess significance between treatment groups. One way ANOVA with post-test using the Tukey-Kramer multiple comparison tests was performed as to determine significance between treatment groups at the confidence intervals indicated. Pair-wise comparisons were performed using unpaired students *t*-test.

### Immunofluorescence assay

Cortical cultures were prepared for microscopy by first rinsing the cultures with phosphate-buffered saline (PBS), followed by fixing the cells with freshly prepared 2% paraformaldehyde in PBS. Following five rinses with PBS (minimum of 2 minutes per rinse), cells were permeabilized with 0.2% triton-X 100 in PBS, rinsed (3 rinses with PBS, minimum of 5 minutes per rinse) and blocked with Image-it FX signal enhancer solution (Molecular Probes). Primary antibodies were added at dilutions of (1:500) for rabbit polyclonal antibody for EP1 (final concentration 1 μg/ml), (1:400) mouse monoclonal antibody for MAP-2 (final concentration 1 μg/ml), (1:500) rat monoclonal GFAP (final concentration of 1 μg/ml), (1:100) rat polyclonal for F4/80 (final concentration 10 μg/ml) and (1:100) rat monoclonal antibody for CD11b (final concentration 5 μg/ml). Incubation of the anti-EP1 antibodies with the EP1 epitope blocking peptide (10:1 molar ratio of peptide: antibody) (Cayman Chemical Company) blocked binding (data not shown). Secondary antibodies were diluted (1:400) for FITC-conjugated anti-mouse (IgG, H&L) (final concentration 3 μg/ml), (1:400) FITC-conjugated anti-rat (final concentration 3 μg/ml), and (1:800) Cy5-conjugated anti-rabbit (IgG, H&L) (final concentration 1.5 μg/ml). The fluorochrome-conjugated antibodies were cross-adsorbed to immunoglobulins from other species to ensure specific multiple labeling. All antibodies were diluted in PBS with 0.025% Tween 20. Combinations of the selective primary antibodies were added to the sections and incubated overnight in a humidified chamber at 4°C. Conjugated secondary antibodies were added for 1 hour at room temperature (RT) followed by staining with PI to visualize cell nuclei. PBS washes were done (3 rinses with PBS, minimum of 5 minutes per rinse) between measures prior to mounting. Sections were mounted with ProLong Gold anti-fade permanent mounting media (Molecular Probes). To account for any background non-specific staining, negative controls with normal mouse and normal rabbit serum at concentrations comparable to primary antibodies were conducted in parallel.

### Confocal laser scanning microscopy

Personal Confocal Microscope PCM-2000 (NIKON, Melville, NY) with Argon, HeNe green and HeNe red lasers were used to acquire the images. Simple Personal Confocal Image program (Simple PCI, Compix, Cranberry Township, PA) was used to acquire digital images and construct compilations. The fluorochromes were resolved into three different image channels with respective emission filters. The FITC label was detected with the Argon laser at 488 nm, Cy5 with the red HeNe laser at 633 nm and PI was visualized with the green HeNe laser at 605/32 nm narrow band pass width filter. The stained cultures were individually scanned for each respective fluorochrome and the multi-focal program (z-focus) was used to create a stereopsis image. The three separate images were merged together to acquire the final triple-colored image; the PI image was converted to blue color during merge. This allowed for comprehensive visualization of labeling throughout the entire depth of the sample. Images were stored as digital files and the final figures were created using Photoshop (Adobe, San Jose, CA). The total magnification of each image presented is equal to (magnification of the objective) × (width of the image panel in cm) × (0.93). This scale of image size was based on size calibration using an American Optical improved Neubauer hemacytometer and simple PCI linear calibration scales.

### Quantification of neuronal nuclear EP1 expression in different culture preparations

The amount of neuronal expression in the nucleus was quantified from digital images of immunofluorescence confocal microscopy of cortical cultures stained with PI (for nuclei) and immuno-stained for EP1, and MAP-2. Each image was captured with the same sensitivity settings on the confocal microscope and all images were captured with a 60× objective with 640 × 480 pixels. A blinded observer (unaware of the culture preparation) counted the number of pixels over the nuclear regions of the neurons that were stained for EP1 with an enlarged image with Adobe Photoshop. Each culture preparation was examined in four independent fields and the number of neurons analyzed varied from 50–70 for each culture preparation.

### Prostaglandin measurements

PGE2 was measured using a prostaglandin enzyme-linked immunosorbent assay (ELISA) detection kit (Cayman Chemical Company) that uses a PGE2-specific antibody. For both the mixed cultures (15 DIV) and neuronal-enriched cultures (8 DIV), the media was exchanged from the standard growth media (MEM with Earle's salts/10% horse serum, 30 mM glucose, 2 mM glutamine) to HBSS+ (Mg^2+^-free Hanks buffered salt solution, 3 mM CaCl_2_, 20 mM HEPES (pH 7.55), 13.4 mM NaHCO3, 30 mM glucose and 25 μM glycine). Since the neuronal-enriched cultures did not survive a direct media exchange, the media was exchanged in the 12-well plates by seven two-fold dilutions (repeated series of addition of 1 ml of HBSS+ to 1 ml of media, followed by removal of 1 ml). This resulted in 0.78% of the original growth media remaining which had no detectable amounts of PGE2. After the media was exchanged, the cultures were exposed to a treatment of NMDA that yielded < 10% neuronal death over the 90 min of the experiment. The mixed cultures were treated with 100 μM NMDA for 15 min followed by treatment with 3 μM of the NMDA antagonist MK801. The neuronal-enriched cultures were treated with 200 μM NMDA for 20 min, followed by treatment with 3 μM MK801. The media was removed from the cultures 90 minutes after the initial NMDA treatment and immediately frozen (-80°C) for analyses at a later time. The amount of PGE2 in the media was determined by enzyme immunoassay as per the instructions from the manufacturer. This assay can detect as little as 8 pg/ml of PGE2.

## Results

### Neuroprotection by an EP1 receptor antagonist

**Figure 2:**
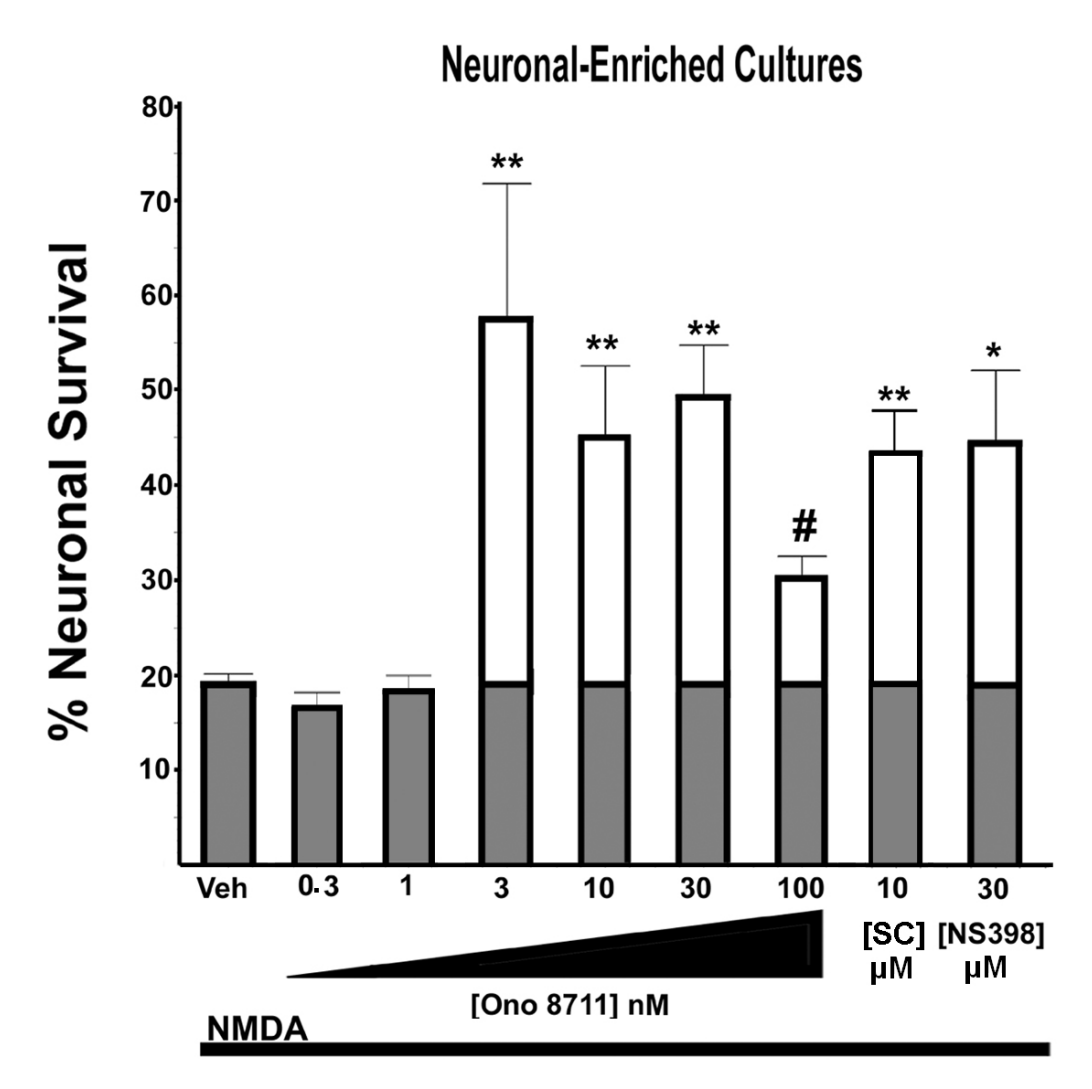
**Neuroprotection by EP1 receptor antagonists and a COX-2 inhibitor in neuronal-enriched cultures**. The effect of varying concentrations of Ono 8711 on neuronal survival to NMDA treatment was assessed using the assay outlined in Figure 1 (see methods). Cultures were either pretreated with the drug vehicle DMSO (veh), or the concentration of the drug indicated below each bar. The cultures were then treated with a concentration of NMDA that leaves only about 20% survival in the non-drug treated cultures (average [NMDA] = 15 μM). The percent survival for the treatments indicated was determined and plotted as percent surviving neurons. The shaded region of the bar graph shows the survival obtained with NMDA alone and the unshaded portion of the bar indicates the additional neuronal survival resulting from co-treatment with the drug indicated. In each experiment, the neuronal cell numbers were counted from four fields (similar to that shown in Figure 1) from each treatment and averaged. The results shown were then average from at least four independent experiments. (* indicates *P *< 0.05 and ** indicates *P *< 0.01 in comparison to vehicle treatment, # indicates a < 0.05 in comparison to the concentrations (3,10 and 30 nM) of Ono 8711, by ANOVA, Tukey-Kramer multiple comparison test). The 100 nM Ono 8711 was not significantly different from the vehicle control. Error bars are standard error of the mean (SEM).

The neuroprotective effect of the EP1 receptor antagonist Ono 8711 was examined using the assay for excitotoxicity shown in Figure 1. The same field of the neuronal culture was photographed before treatments, and twenty hours after continuous exposure with NMDA. In this assay the fate of each neuron can then be followed over time. In Figure 1A, the neuronal-enriched cultures were treated with a concentration of NMDA that leaves only about 20% of the neurons viable as determined by trypan blue exclusion (Figure 1C). However, when 30 nM Ono 8711 was added to a sister culture prior to treatment with the same concentration of NMDA, there was a dramatic increase in the number of surviving neurons (Figure 1B and 1D). A more extensive analysis using this assay was performed with treatments of Ono 8711 at concentrations of 0.3 nM, 1 nM, 3 nM, 10 nM, 30 nM and 100 nM added just prior to treatment with NMDA. As seen in Figure 2, treatment with as little as 3 nM of Ono 8711 increased the percent of surviving neurons to nearly 60%, compared with 20% as seen with NMDA alone. Similar amounts of neuroprotection were observed with treatments of 10 nM and 30 nM Ono 8711 and were not significantly different than treatments with 30 μM of the COX-2 inhibitor NS398. This concentration of NS398 is the optimal concentration for protection with this drug [2,3]. However, the highest concentration of Ono 8711 tested (100 nM) yielded no significant neuroprotection and resulted in significantly lower amounts of surviving neurons than observed with 3, 10 and 30 nM Ono 8711. Treatment of cultures with 100 nM Ono 8711 in the absence of NMDA did not result in any detectable toxicity of neurons (data not shown). Another EP1 receptor antagonist, SC51809 [28] at a concentration of 10 μM, produced the same amount of neuroprotection in neuronal-enriched cultures as seen with Ono 8711 and 30 μM NS398 (Figure 2).

### Neuroprotection by EP1 antagonists is sensitive to modulation by non-neuronal cells

**Figure 3:**
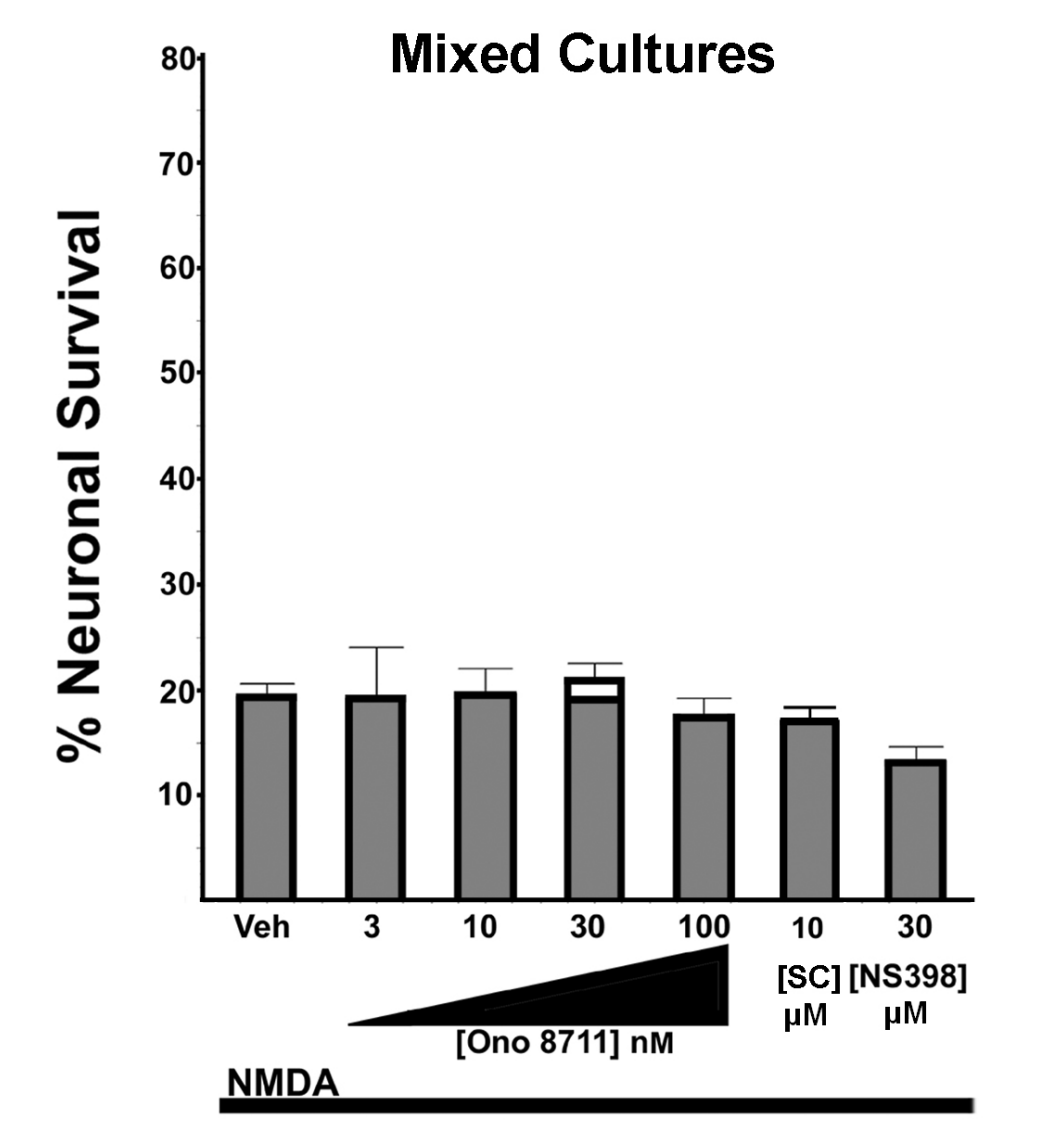
**Lack of neuroprotection by EP1 receptor antagonists or NS398 in mixed cultures**. Ono 8711, SC51089 and NS398 did not confer neuroprotection in mixed cultures. Mixed cortical cultures were treated with the concentrations of Ono 8711 indicated, 10 μM SC51089 or 30 μM NS398 followed by treatment with NMDA (average [NMDA] = 20 μM). These cultures were treated in parallel with the neuronal-enriched cultures shown in Figure 2. Neuroprotection was not observed with any concentration of Ono 8711, SC51089, or NS398. The results shown were the average (+SEM) from four independent experiments.

In the preceding experiments, the neuroprotective effects of Ono 8711 and SC51089 were assessed using neuronal-enriched cultures where neurons account for about 90–95% of the cells in the culture (see methods). Neuroprotection mediated by the EP1 antagonists in these neuronal-enriched cultures, is consistent with EP1 antagonists directly blocking EP1 receptor activation on neurons. In order to assess whether this effect is modulated by other cell types, we examined whether EP1 receptor antagonists were neuroprotective in a mixed culture preparation, where approximately 30% of the cells present in the culture were neurons (see methods). In sharp contrast to the results obtained with neuronal-enriched cultures, there was no appreciable neuroprotective effect elicited by treatment with any concentration of the EP1 antagonist Ono 8711, 10 μM SC51089 or with 30 μM NS398 (see Figure 3). A similar lack of neuroprotection by the EP1 antagonists was also observed when neuronal-enriched cultures were prepared with insufficient amounts of the anti-mitotic agent AraC (see methods) to limit the growth of non-neuronal cells (data not shown).

### Media exchange between cultures

**Figure 4:**
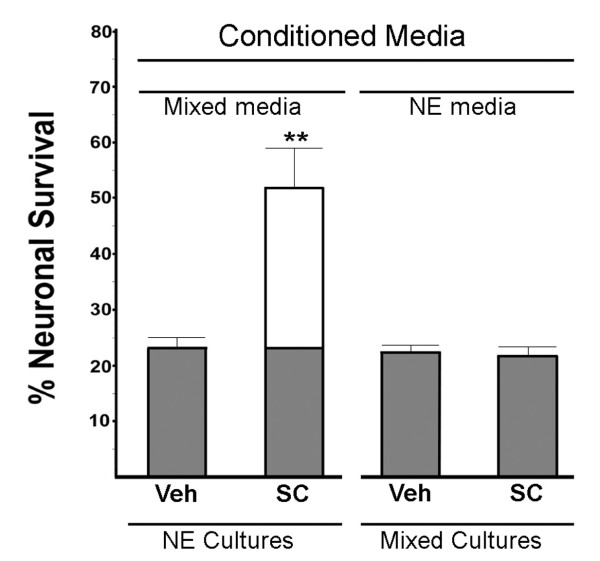
**Neuroprotection following media exchange between mixed and neuronal-enriched cultures**. Media from mixed and neuronal-enriched (NE) cultures was exchanged (see methods) prior to challenge with NMDA (average [NMDA] = 15 μM for NE cultures and 20 μM for mixed cultures) in the presence or absence of the EP1 antagonist SC51089 (shown as SC). The source of the media is shown above the bars and the culture below the bars. This is the average of four independent experiments. The protection by SC in the neuronal-enriched cultures with mixed media was significant (*P *< 0.01) by ANOVA, Tukey-Kramer multiple comparison test. The results shown were then averaged from four independent experiments.

To assess whether the difference in response to the EP1 antagonists was due to differences in cell-derived components in the culture media, experiments were done where the media was exchanged between the two cultures. As seen in Figure 4, EP1 antagonist SC51089 was still neuroprotective in the neuronal-enriched cultures with media transferred from mixed cultures. Conversely, SC51089 still did not confer neuroprotection in mixed cultures containing media from neuronal-enriched cultures. Therefore, the neuroprotective response to SC51089 remained the same, irrespective to the source of media. These results illustrate that the cellular environment in the mixed cultures is important to determine whether the EP1 antagonist is neuroprotective. Alternatively, components in the media from the mixed cell cultures are labile and lose activity during the media transfer procedure or require longer exposure time to elicit an effect.

### Cellular composition of mixed and neuronal-enriched cultures

**Figure 5:**
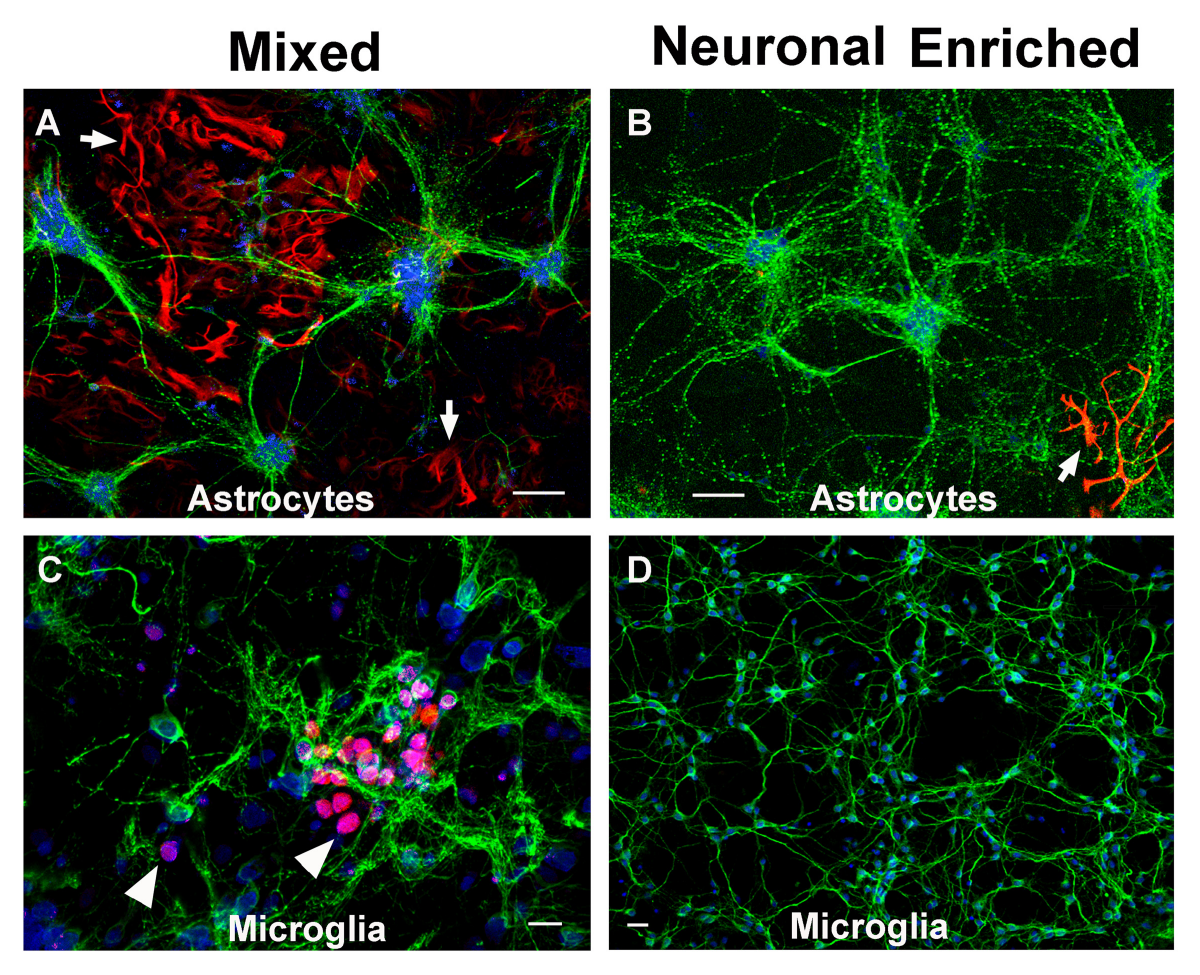
**Cellular composition of mixed and neuronal-enriched cultures**. Confocal immunofluorescence microscopy was performed on mixed and neuronal-enriched cultures that were stained with neuronal specific antibody MAP-2 (green) and either the astrocyte-specific marker GFAP (red) (A and B) or the microglia marker F4/80 (red) (C and D), nuclei were stained with PI and appear blue. Prominent GFAP staining in mixed cultures is indicated with an arrow (A) and the rare astrocyte in the neuronal-enriched culture is shown with an arrow (B). Microglia were prominent in mixed cultures (C) and two examples were marked with an arrow. Microglial staining could not be detected in the neuronal-enriched cultures (D). Images were captured at 10× magnification for A and B, 60× for C and 40× for D. Magnification bars are 100 μm for A and B, 20 μm for C and D.

Cellular composition in both mixed and neuronal-enriched cultures was examined by confocal immunofluorescence microscopy to identify possible candidate non-neuronal cell types that influence EP1 neuronal response. Mixed cultures contained an abundance of cells stained with the astrocyte-specific marker GFAP (Figure 5a) in contrast to the neuronal-enriched cultures which contained a rare GFAP-positive cell (Figure 5b). Microglia were also present in the mixed cultures as indicated by the presence of cells that contain the monocyte/macrophage lineage marker, F4/80 (Figure 5c). F4/80 positive cells were not detected in the neuronal-enriched cultures (Figure 5d). Similar results were obtained with the microglial marker, CD11b (not shown). These results identify both astrocytes and microglia as potential cell types in mixed cultures that could negate the neuroprotective effects of EP1 antagonists.

### Transwell co-cultures with neuronal-enriched cultures

**Figure 6:**
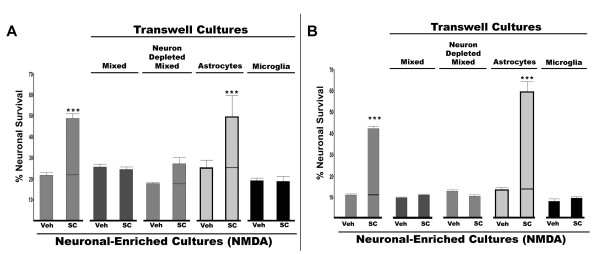
**Effects of transwell co-cultures on EP1 antagonist-mediated neuroprotection**. Transwells containing either mixed cultures, neuron-depleted mixed cultures, astrocytes or microglia cultures were co-cultured with neuronal-enriched cultures for 48 hours prior to treatment with vehicle (veh) or the EP1 antagonist SC51089 (SC) and subsequent challenge with NMDA (average [NMDA] = 15 μM for neuronal-enriched cultures and astrocyte-containing cultures and 16 μM for cultures with transwells containing mixed cultures, neuron-depleated mixed cultures and microglia). Neuronal viability was assessed 20 hours after NMDA in each culture paradigm (as outlined in Figure 1) and compared to neuronal-enriched cultures without transwells (first two bars in each set). The composition of the transwell cultures are marked above the bars. The relative amount of neuroprotection was assessed with two different amounts of NMDA-induced excitotoxicity 20% survival (A) and 10% survival for (B). For 10% survival the average [NMDA] = 12 μM for neuronal-enriched cultures and astrocyte-containing cultures and 18 μM for cultures with transwells containing mixed cultures, neuron-depleated mixed cultures and microglia. Each set is the average (+ SEM) of four independent experiments. Protection by SC in neuronal-enriched cultures with and without astrocytes was highly significant (*P *< 0.001 by ANOVA Tukey-Kramer multiple comparison test). A line is included in the bar to show the additional amount of survival conferred by addition of the EP1 antagonist.

In order to assess which of these different cell types could affect neuroprotection by an EP1 antagonist, a series of experiments were performed where different types of cultures were grown on permeable transwell filters and placed above neuronal-enriched cultures. When transwells containing mixed cultures were placed above the neuronal-enriched cultures (during and 48 hours prior to NMDA challenge), the EP1 antagonist was not neuroprotective in the neuronal-enriched cultures grown below (Figure 6). This experimental system with the mixed culture transwells now made the neurons in the neuronal-enriched cultures respond similarly to EP1 antagonist as the neurons contained in the mixed cultures (Figure 3). This illustrates that soluble factors from the mixed cultures influenced how the neurons in the neuronal-enriched cultures responded to EP1 antagonists. Mixed cultures devoid of neurons also produced the same effect when placed on transwells above neuronal-enriched cultures rendering the neurons in the neuronal-enriched cultures insensitive to the neuroprotective effects of the EP1 antagonist (Figure 6). This suggests that non neuronal cells in the mixed cultures were negating the neuroprotective effect of the EP1 antagonist.

The effects of astrocytes and microglia were then individually examined for their potential influence on the neuroprotective effect of the EP1 antagonist. Astrocyte transwell cultures appeared to have little effect on the neuroprotection conferred by SC51089. However, when cultures of microglia (90% of the cells were positive for the microglial marker, F4/80) grown on transwells were placed above the neuronal-enriched cultures, SC51089 was not neuroprotective (Figure 6). These same trends for the different combinations of transwell cultures on neuronal-enriched cultures were seen at two different levels of NMDA-induced excitotoxicity (20% survival, Figure 6a and 10% survival, Figure, 6b). None of the transwell cultures were toxic to the neurons in the neuronal-enriched cultures in the absence of NMDA (data not shown). Therefore, these experiments showed that the presence of microglia blocks neuroprotection by an EP1 antagonist. The same effects of microglia on neuroprotection by an EP1 antagonist was also observed in cultures grown in defined serum-free media (see methods). In these cultures, microglial transwells also abrogated the neuroprotective effects of SC51089 on neuronal-enriched cultures (data not shown). This indicates that the effect of microglia on neuroprotection by an EP1 antagonist was not due to serum-derived components in the growth media.

### Effects of microglia on sensitivity to NMDA-induced excitotoxicity

**Figure 7:**
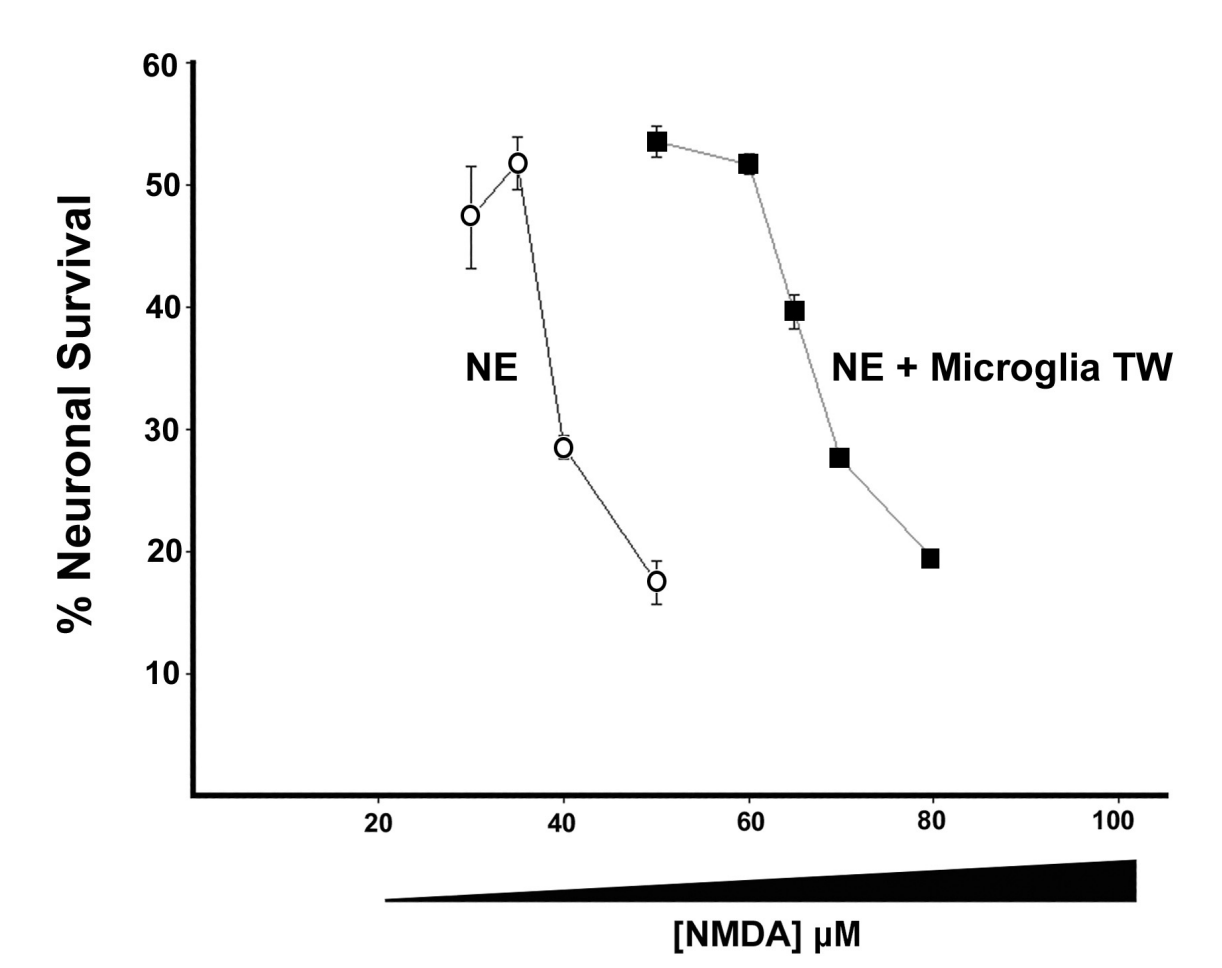
**Microglial influence on NMDA-induced neuronal death**. Neuronal-enriched cultures were incubated with or without microglial transwell cultures for 48 hours and were exposed to varying concentrations of NMDA. Neuronal death was assessed as depicted in Figure 1 after 20 hours of continuous NMDA exposure. The results are the average of two independent experiments. Error bars for each concentration of NMDA represent the SEM.

In the preceding experiments, the relative neuroprotective effects of the EP1 antagonists with and without microglia were compared under conditions where NMDA was adjusted to produce equivalent amounts of neuronal death in each culture condition. However, when microglia were present in transwells above the neuronal-enriched cultures, the neurons were more resistant to NMDA-mediated excitotoxicity as indicated in the concentration-response curves shown in Figure 7. Therefore, in this context, microglia conferred a neuroprotective effect on the cultures. However, despite this neuroprotective effect elicited by microglia, EP1 antagonists lacked any additional neuroprotective effect in cultures containing microglia.

### Microglia alter neuronal response to EP1 antagonists

**Figure 8:**
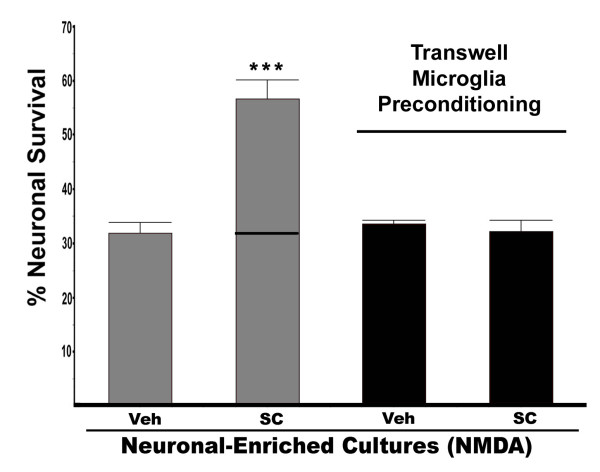
**Microglial influence on EP1 antagonist-mediated neuroprotection**. Neuronal-enriched cultures were cultured in the absence or presence of microglia-containing transwells for 48 hours after which the microglial transwells were removed (pre-conditioning with microglia). The cultures were then treated with NMDA (average [NMDA] = 25 μM for neuronal-enriched cultures and 100 μM for microglia preconditioned media) in the presence or absence of the EP1 antagonist SC51089 (10 μM) and scored for neuronal survival 20 hours later. Neuroprotection is only seen in the neuronal-enriched cultures not exposed to microglia (*P *< 0.001 ANOVA Tukey-Kramer multiple comparisons test). A line is included in the bar to show the additional amount of survival conferred by addition of the EP1 antagonist. These results show that preconditioning with microglia for 48 hours prior to NMDA treatment is sufficient to inhibit the neuroprotective effect of the EP1 antagonist. This is the average of three independent experiments.

The preceding experiments demonstrated that the presence of microglia in the culture alter how the EP1 receptor antagonists confer neuroprotection following an excitotoxic challenge with NMDA. However, those experiments do not distinguish whether these differences are due to microglial-induced changes in neuronal EP1 response or are an indirect consequence of EP1 signaling in the microglia. In order to test whether microglia can induce changes in neuronal response to EP1 antagonists, neuronal-enriched cultures were co-cultured with microglial transwells for 48 hours, then the microglial transwells were removed prior to challenge with NMDA. As seen in Figure 8, this pre-incubation with microglia was sufficient to abolish the neuroprotective affect of the EP1 antagonist in the neuronal-enriched cultures. Notably, the microglial preconditioned neuronal-enriched cultures were also more resistant to NMDA similar to that seen when microglia are present during the excitotoxic challenge (data not shown). Since microglia need not be present during the challenge with NMDA to alter the neuroprotective effects of EP1 antagonist, this would suggest that microglia are able to alter the EP1 response in neurons. The potential effect of microglia on EP1 expression and NMDA induced PGE2 production was subsequently examined.

### Neuronal expression of EP1 in mixed cultures and neuronal-enriched cultures with and without microglial transwells

**Figure 9:**
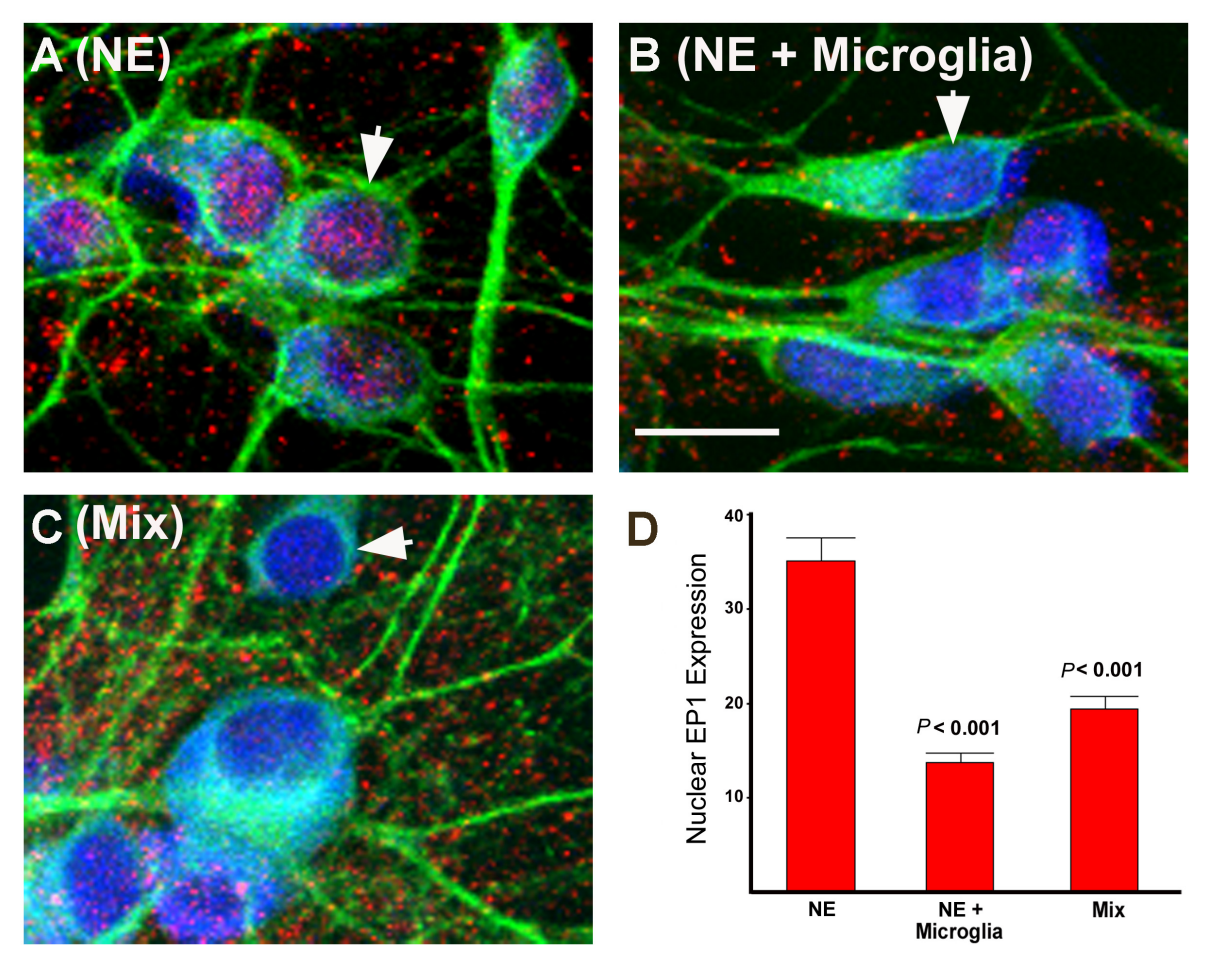
**Neuronal expression of EP1 receptor in cultures with or without microglia**. Neuronal-enriched cultures (A), neuronal-enriched cultures with microglia (B) and mixed cultures (C) were immunostained with antibodies against the neuronal-specific marker, MAP-2 (green), EP1 (red) and nuclei were stained with propidium iodide (PI) (blue) prior to confocal microscopy. Nuclei with different amounts of EP1 staining are indicated with arrows in each panel. All images were captured at 60× magnification and the bar indicates 10 microns. The relative amounts (+SEM) of nuclear EP1 expression for neuronal-enriched (NE), NE + microglia, and mixed cultures was determined for 60–75 neurons for each condition (see methods) and plotted in panel D. The neuronal-enriched cultures lacking microglia had significantly higher amounts of nuclear EP1 expression (ANOVA Tukey-Kramer multiple comparison test).

EP1 receptor antagonists may have diverse neuroprotective properties with different culture preparations because of potential microglial-induced changes in neuronal EP1 receptor expression. In order to address this possibility, the neuronal expression of EP1 receptor was examined by immunofluorescence confocal microcopy for mixed cultures and neuronal-enriched cultures in the presence or absence of microglial transwells. As seen in Figure 9, there was no appreciable net reduction in the amount of EP1 receptor expressed in microglia-containing cultures. However, there was a significant, two-fold decrease of EP1 expression in the nucleus in the neuronal-enriched cultures containing microglial transwells (Figure 9b) and in the mixed cultures (Figure 9c) compared to neuronal-enriched cultures (Figure 9a). Therefore, higher amounts of nuclear EP1 were seen in the cultures in which EP1 antagonists were neuroprotective.

### NMDA stimulation of PGE2 in mixed and neuronal-enriched cultures

**Figure 10:**
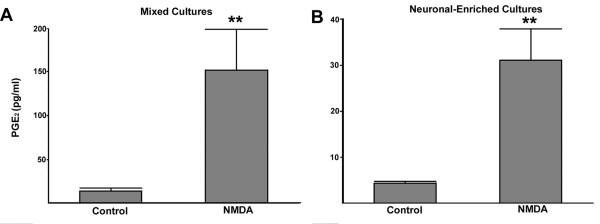
**NMDA induces prostaglandin E2 production in mixed and neuronal-enriched cultures**. Media from mixed (A) and neuronal-enriched (B) cultures were analyzed by ELISA for the presence of PGE2 from cultures either treated with NMDA or vehicle (control). The amount of PGE2 detected (pg/ml) is plotted for the average (+SEM) from 4 independent experiments. Note that the scales of PGE2 differ between preparations. (**indicates *P *= 0.01 in comparison to vehicle treatment by ANOVA, Tukey-Kramer multiple comparison test).

We have shown previously that NMDA stimulates the synthesis and release of PGE2 in cortical cultures [3,14]. A comparison of the NMDA-stimulated release of PGE2 by neuronal-enriched and mixed cultures was done to assess whether differences in neuroprotection by COX-2 inhibitors and EP1 receptor antagonists could be due to changes in PGE2 production. Both culture preparations showed about a six to nine-fold increase in PGE2, 90 minutes after treatment with NMDA (Figure 10A and 10B) and this induction was blocked by the NMDA receptor antagonist MK801 (3 μM) (data not shown). The net amount of PGE2 in the mixed cultures was about five-fold higher than in the neuronal-enriched cultures. In both cases the concentration of NMDA-induced PGE2 (425 pM for mix and 85 pM for neuronal-enriched cultures) was below the Ki reported for the EP1 receptor of 22 nM [33]. However, as noted previously [14], the concentration of PGE2 in the media may not reflect higher local concentrations at the site of synthesis near the neurons. These findings indicate that a lack of neuroprotection with the EP1 antagonists in cultures containing microglia was not due to an inability to synthesize PGE2.

## Discussion

In this study we found that two structurally distinct EP1 receptor antagonists (Ono 8711 and SC51089) protect a significant percentage of neurons against a sustained exposure to NMDA in primary neuronal cultures devoid of microglia. The neuroprotective effect of these agents is mediated by specifically blocking the EP1 receptor since maximal neuroprotection with Ono 8711 occurred with concentrations as low at 3 nM, which is just above the Ki for the EP1 receptor (1.7 nM), (Watanabe et al., 1999). Concentrations of Ono 8711 greater than 100 nM were not directly toxic to neurons, but had no significant neuroprotective effect agonist NMDA-mediated toxicity. It is not known what accounts for this effect, but higher concentrations of Ono 8711 may have off target actions which may alter the contribution of EP1 to neuronal viability or neuronal sensitivity to excitotoxic stimuli.

The pharmacological evidence that we demonstrated with EP1 receptor antagonists is consistent with recent in vitro [21,22] and in vivo studies demonstrating that EP1 activation contributes to neuronal death [23,24]. The role that neuronal EP1 activation plays in augmentation of neuronal excitotoxicity is now beginning to be elucidated. EP1 is a G-protein coupled receptor linked to the inositol triphosphate (IP3) signaling cascade and causes release of intracellular calcium [34]. Recent studies by Iadecola and colleagues demonstrate that EP1 activation impairs the Na^+^-Ca^2+ ^exchanger (NCX) leading to dysregulation of intracellular calcium following NMDA receptor stimulation [23]. The resulting increase in intracellular cytoplasmic calcium concentration is an integral component to excitotoxic death and contributes to neuronal death [35,36]. Another recent study indicated that EP1 activation can also contribute to excitotoxic death through the AKT/PKB (protein kinase B) signaling cascade [26]. NMDA-induced neuronal death is associated with decreased AKT excitotoxic stimuli [37]. EP1 inhibitors increase AKT activity by inactivating the inhibitor of AKT, PTEN (phosphatase and tensin homologue deleted on chromosome 10), and subsequently inhibiting translocation of the proapoptotic protein BAD [26]. Our finding that EP1 antagonists protected neurons in neuronal-enriched cultures is also consistent with a mechanism in which neuronal activation of EP1 leads to cell death.

Our results indicated that when microglia were present in the cultures, the neuroprotective properties of EP1 antagonists were lost. Gendron et al. demonstrated that EP1 antagonists conferred neuroprotection against excitotoxicity induced by oxygen-glucose-deprivation in mixed neuronal cultures [22]. However, they did not characterize the cellular composition with respect to microglia. In their culture system anti-mitotic agents were added to their mixed cultures on day 4 after plating compared to our mixed cultures in which the anti-mitotic agents were added on day 9 (see methods). This difference may have limited the amounts of microglia in their system.

In our culture system, the loss of neuroprotection with the EP1 antagonist occurred when the microglia were either cultured with the neurons (as in the mixed cultures) or when the microglia were co-cultured in transwells above the neuronal-enriched cultures. Co-culturing of neuronal-enriched cultures with microglia for 48 hours was also sufficient to alter the neuroprotective effects of EP1 antagonists. This indicates that the microglial cultures were able to produce soluble factors that could diffuse through the 0.4 μm pores in the transwell membrane and influence EP1 response in the neurons in the neuronal-enriched cultures. Simple transfer of media from the mixed cultures to the neuronal-enriched cultures could not abolish neuroprotection. This indicates that the soluble component(s) from the mixed cultures that were able to affect EP1 response in the transwell cultures are either labile, not incubated for sufficient times before NMDA challenge or are produced in the mixed cultures during NMDA stimulation. Identification of these components produced by microglia and how they modulate neuronal EP1 response will require further investigation.

The mechanism by which microglia inhibit the neuroprotective effects of EP1 antagonists appeared to be more complex than just downregulation of neuronal EP1 expression or decreased NMDA-induced synthesis of PGE2. However, there was a significant difference in cellular distribution of EP1 to the nuclear region in neurons from cultures that lacked microglia. Additional studies will be required to determine whether this change in EP1 localization is important to EP1 signaling and the viability of neurons following NMDA stimulation. Nuclear localization of EP1 has been observed in neurons and brain-derived endothelial cells, where stimulation of EP1 receptors can induce changes in nuclear calcium concentrations and modulate gene expression of genes such as *c-fos *[38].

In our culture systems, neurons were more resistant to NMDA induced excitotoxicity when microglia were present. Decreased neuronal nuclear expression of EP1 was also observed in microglia-containing cultures. It is possible that the neuroprotective effect of microglia could be in part due to a redistribution of EP1 expression.

Our data support a role for microglia in altering the neuronal EP1 response. However, we cannot rule out whether EP1 signaling in microglia can alter neuronal viability. Further studies will be needed to assess the role that EP1 signaling in microglia play in modulating neuronal viability. EP1 receptors were abundantly expressed in microglia in our cultures (data not shown) and could play some role in the interaction with neurons.

This study identified a new role for microglia as an important modulator of the neuroprotective properties of EP1 antagonists in vitro. If the same interactions were to occur in vivo, this could have important consequences with respect to microglial-mediated effects on neuronal viability. In vivo, microglia migrate to the site of neuronal injury and have been implicated to play a role in stroke and neurodegenerative diseases such as Alzheimer's disease, Parkinson's disease, multiple sclerosis, and HIV-dementia [39]. It is presently controversial whether microglia play a harmful or beneficial role in neuronal death [39-41]. The role played by microglia in neuronal death may differ depending on the stimuli used to induce death, the cellular milieu and the presence of various factors along with the specific intracellular pathways that are activated [39].

There are two examples in vitro where microglia have been shown to be detrimental towards neurotoxin-induced neuronal death that is associated with COX-2. In an in vitro model of Parkinson's disease, COX-2 inhibitors showed a dramatically reduced level of neuroprotection towards dopaminergic neurons in cultures that lack microglia following MPP^+ ^(1-methyl-4-phenylpyridinium)-induced death [42]. Microglia were shown to be the major source of MPP^+^-induced production of PGE_2 _as well as contribute to increasing neuronal COX-2 induction. In another example, microglia are also required for lipopolysaccharide (LPS) – induced neuronal death in culture [43]. In this case, stimulation of the EP2 receptor on microglia induced expression of COX-2 and iNOS which were required for neuronal death. These examples differ from our studies where microglia appeared to have neuroprotective effects and diminished the contribution of neuronal EP1 receptor activation to excitotoxic death.

In light of the recently identified complications of cardiovascular disease associated with COX-2 inhibitors ([25,44,45], it is important to identify new targets down-stream of COX-2 that contribute to neuronal death [46]. Inhibition of EP1 represents an attractive alternative to COX-2 inhibition for neuroprotective strategies that could be used for treating stroke and other neurodegenerative diseases [23,24,47,48].

## Conclusion

This study demonstrates that microglia may limit the contribution of EP1 receptor activation towards neuronal excitotoxicity. Understanding how microglia could limit the contribution of EP1 to neuronal excitotoxicity is not only an important consideration for designing neuroprotective treatments with EP1 antagonists but may also generate new approaches in the treatment of neurodegenerative disease.

## Competing interests

The authors declare that they have no competing interests.

## Authors' contributions

NGC is the major contributor in drafting the manuscript and revising it critically for important intellectual content, conception and design, acquisition of data, analysis and interpretation of data. MAR has played a major role in revising the manuscript critically for important intellectual content conception and design, acquisition of data, analysis and interpretation of data. J-DB has also made significant contribution to design, acquisition of data, analysis and interpretation of data. JWR, JH and KEH played important roles in design and acquisition of data, analysis and interpretation of data. JWR has have been involved in drafting the manuscript and revising it critically for important intellectual content, conception and design. All authors read and approved the final manuscript.
